# Insights into human respiratory microbiome under dysbiosis and its analysis tool

**DOI:** 10.3389/frmbi.2025.1549166

**Published:** 2025-04-28

**Authors:** Mehfooz Helal, Vinay Kumar Bari

**Affiliations:** Department of Biochemistry, School of Basic Sciences, Central University of Punjab, Bathinda, India

**Keywords:** microbiota, respiratory tract, next generation sequencing, omics tools, dysbiosis

## Abstract

The human respiratory tract microbiome is a multi-kingdom microbial ecology that inhabits several habitats along the respiratory tract. The respiratory tract microbiome promotes host health by strengthening the immune system and avoiding pathogen infection. The lung microbiome mostly originates in the upper respiratory tract. The balance between microbial immigration and removal determines the nature of the lung microbiome. Identification and characterization of microbial communities from airways have been made much easier by recent developments in amplicon and shotgun metagenomic sequencing and data analysis techniques. In pulmonary medicine, there is a growing interest in the respiratory microbiome, which has been linked to human health and illness. However, the primary causes of bacterial co-occurrence seem to be interactions with fungi and bacteria as well as host and environmental factors. This study focused on identifying techniques and the current understanding of the relationship between the microbiota and various lung diseases.

## 1 Introduction

The respiratory microbiome is a heterogeneous system of microbes that includes bacteria (bacteriome), fungi (mycobiome), viruses, and phages (Schenck et al., 2016; Man et al., 2017). The host’s immune response tolerates members of the microbiome in a symbiotic relationship with the host (human). In a healthy individual, bacteria represent the highly diverse and dominating member of the microbiome, but the microbiome composition may change when the immune system is compromised (Belkaid and Hand, 2014; Tomkovich and Jobin, 2016; Budden and Romani, 2025). Microorganisms create unique microenvironments, leading to variations in the microbiome at finer taxonomic levels in different regions. The makeup of these microbial communities is shaped by environmental conditions and the interactions between microbes and the host’s immune system (Huffnagle et al., 2017). The normal microbiota plays a protective role by preventing the establishment of harmful pathogens. It competes with pathogens for attachment sites and nutrients, making it challenging for them to establish, multiply, and cause disease. However, some microorganisms can cause infection when given a condition of weakened immune response or when antibiotics are used (Kumpitsch et al., 2019).

With the advent of next-generation sequencing (NGS) technology, it has been revealed that in bacteriome of the healthy human respiratory system, *Firmicutes* and *Bacteriodetes* represent the most common phyla found in the respiratory system, with *Prevotella, Streptococcus*, and *Veillonella* being the most common genera (Whiteside et al., 2021). Most fungi detected in the human respiratory system belong to the Basidiomycota and Ascomycota. The most prevalent genera in lung tissue include *Cladosporium*, *Eurotium*, and *Aspergillus* (Charlson et al., 2012). However, culture-independent characterization using 16S/18S rRNA gene sequence has thoroughly revealed the upper respiratory tract (URT) microbiome; the lower respiratory tract (LRT) microbiome has not been studied much (Segal and Blaser, 2014). The role of gut commensals in health and disease has been extensively researched. However, there is relatively little information available about the commensals residing in the respiratory tract, which contains a diverse microbial community that is constantly exposed to external environmental influences including COVID-19 (Emadi et al., 2024a). *Aspergillus*, *Penicillium*, and *Candida* genera are the main pulmonary fungal pathogens in patients with respiratory diseases, but several ecological species, such as *Cladosporium cladosporioides* and *Eremothecium sinecaudum*, are also present in the lung mycobiome of healthy persons (Pendleton et al., 2017). The genus *Malassezia* is overrepresented in cystic fibrosis (CF) and asthma patients (Nguyen et al., 2015). In individuals with severe Chronic obstructive pulmonary disease (COPD), *Aspergillus* species are shown to be highly prevalent (Saiman and Siegel, 2004; Huerta et al., 2014), while *Candida* spp., has been found in idiopathic pulmonary fibrosis (IPF) patients (Cuerpo et al., 2019).

## 2 Upper respiratory tract microbiome

The upper respiratory system comprises a network of cavities consisting of the nostril, oropharynx, and rhino-pharynx, communicating with the larynx via the Eustachian tube (Morris, 1988). The unique properties of every anatomical location in this system, including temperature, humidity, oxygen availability, and the kind of epithelial cells lining these structures, produce distinct microenvironments that eventually influence the variety of the microbiome. As a result, the microbiome varies at different taxonomic levels in each region. The interaction between the host immune system and the microbes also influences the microbiome’s composition (Lambring et al., 2019; Zheng et al., 2020). Generally, the normal resident microbiota restricts the growth of harmful pathogens by limiting access to nutrients as well as indirectly by strengthening host innate and adaptive immune responses; hence, pathogens face competition for attachment sites and nutrients, making it harder for them to establish themselves, multiply, and cause disease. This competition among microbes is also a key factor in shaping the overall microbiome (McKenney and Kendall, 2016). The colonization of the upper respiratory tract by microbiome starts at birth, and the relationship between the initial microbiome and a person’s health is shaped over their lifetime through three main factors: (a) the method of delivery (whether by cesarean section or natural birth); (b) environmental influences (such as living conditions and diet); and (c) the use of antibiotics (Cicinelli et al., 2012; Gallacher and Kotecha, 2016). In the first few hours after birth, healthy full-term newborns harbor a substantial number of microbes in their respiratory tract, most of which are thought to come from the mother. During the initial week of life, the upper respiratory tract has been found to be colonized mainly by microbial genera such as *Corynebacterium*, *Staphylococcus*, and *Dolosigranulum* (Man et al., 2017; Kumpitsch et al., 2019). As time progresses, there is a subsequent rise in the presence of *Moraxella* and *Streptococcus*, and the early presence of *Corynebacterium*, *Dolosigranulum*, and *Moraxella* is strongly associated with good respiratory health (Vissers et al., 2014; Bosch et al., 2016). *Streptococci* is the predominant microbiota in the healthy upper respiratory tract, with *Neisseria, Prevotella, Rothia*, and *Haemophilus* following closely behind (Cui et al., 2013; Kumpitsch et al., 2019). Bacteria that belong to the genera *Actinobacteria, Firmicutes, Proteobacteria*, *Bacteroidetes*, and *Fusobacteria* are the common colonizers of the mucosal surfaces of these structures (Sahin-Yilmaz and Naclerio, 2011). Studies have reported *Corynebacterium, Staphylococcus, Streptococcus, Propionibacterium, Moraxella*, and *Dolosigranulum* as the common colonizer of the nasal cavity and nasopharynx while *Streptococcus, Prevotella, Rothia, Veillonella*, as the common colonizer of the oropharynx. *Candida, Aspergillus*, *Saccharomyces*, and *Penicillium* are the common fungal genera that colonize the URT (Eidi et al., 2016; Campbell et al., 2023).

## 3 Lower respiratory tract microbiome

**Figure 1:**
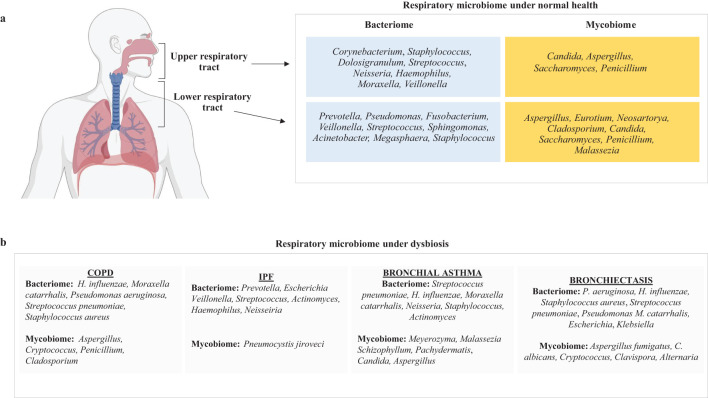
A figure illustrating the key components of respiratory microbiome composition in **(a)** normal healthy humans and **(b)** patients suffering from COPD, IPF, bronchial asthma, and bronchiectasis.

A healthy human’s lower respiratory tract microbiome comprises bacteria, fungi, viruses, and bacteriophages (Li et al., 2024; Lipinksi et al., 2024). The common fungal genera occurring in the lower respiratory tract of an adult human include *Aspergillus, Penicillium, Eurotium, Cladosporium, Malassezia, Candida, Saccharomyces, and Neosartorya* (Pulvirenti et al., 2019). Bacterial genera of LRT include *Prevotella, Fusobacterium, Streptococcus*, *Pseudomonas, Acinetobacter, Megasphaera, Veillonella, Staphylococcus, Sphingomonas* (Dickson et al., 2017). The microbiome in the lungs is not uniform across different parts, such as the bronchi, bronchioles, and alveoli (West, 1978; Dickson et al., 2015). Instead, it is shaped by several factors, including (a) how microbes enter the lungs (such as through inhalation of microorganisms, micro-aspiration, or the spread of mucus), (b) the mechanisms for microbial elimination (like coughing, the body’s innate and adaptive immune responses and mucociliary clearance), and (c) the local environment (including nutrient availability, oxygen levels, temperature, microbial competition, and the presence and activity of inflammatory cells) (Belizário et al., 2023; Natalini et al., 2023). A decline in the lung’s ability to eliminate microbes can enhance conditions for microbial growth, leading to an imbalance in the microbiome, known as dysbiosis, which increases the risk of lung disease (Belizário et al., 2023). It should be noted that the bacteriome and mycobiome always interact with each other within the organ or the tissue in which they are localized (Rozaliyani et al., 2023) (**Figure 1a**).

## 4 Respiratory microbiome and lung diseases

Bacteria constitute most of the respiratory microbiome, while fungi are less prevalent (Zhao et al., 2023). Although mycobiome constitutes a smaller fraction of the total respiratory microbiome, there is significant diversity in the fungal species present in the respiratory tract of individuals (Weaver et al., 2019; de Dios Caballero et al., 2022; Katsoulis et al., 2024). Early research on the sputum microbiome has shown that *Ascomycota* is the dominant fungal group in COPD patients and healthy individuals (Liu et al., 2021). Many lung disorders, including cystic fibrosis, IPF, and COPD, are characterized by microbial dysbiosis, and the advancement of these diseases has been associated with a decrease in the variety of bacterial composition (Dickson et al., 2013; Garcia-Nuñez et al., 2014; Puiu et al., 2024a; Angebault et al., 2025). In this section, we have tried to summarize the respiratory microbiome composition of patients suffering from lung diseases such as COPD, IPF, bronchial asthma, and bronchiectasis (**Figure 1b**).

### 4.1 COPD

Early research on the microbiome of patients with stable COPD showed a strong correlation between the presence of gram-negative enteric bacteria, neutrophilic inflammation, and elevated expression of cytokines like IL-8 and TNF in the lower airways and potentially pathogenic microbes like *Haemophilus influenza*, *Moraxella catarrhalis*, and *Pseudomonas aeruginosa*, *Streptococcus pneumoniae, Staphylococcus aureus* (Soler et al., 1999; Bresser et al., 2000; Sethi et al., 2006; Parameswaran et al., 2009). Bacteria play an important role in the exacerbation of COPD, and it is reported that in nearly 50% of exacerbations, bacteria have been isolated from the lower respiratory tract (Sethi, 2000; Beasley et al., 2012). COPD bacteriome has been reported to vary both spatially and temporally. Changes have been observed in the transitory microbiota during exacerbations as stable COPD and exacerbated COPD microbiota have been reported to differ from each other (Tangedal et al., 2019). Enhancement with *Streptococcus*, *Prevotella*, *Staphylococcus*, and *Gemella* (strains that are part of the normal oral microbiota) was linked to abnormalities in lung function, including a worse response to bronchodiator therapy, in one of a recent study on lower airway microbiome in patients with early COPD (Opron et al., 2021). As COPD worsens, persistent inflammation weakens the innate immune system in the lungs, opening the door for more germs from old and new infections. Numerous studies have demonstrated that there is the enrichment of Gammaproteobacteria including *Moraxella* and *Haemophilus* genera, in moderate to advanced stages of COPD (Pragman et al., 2012; Sze et al., 2012) and also with the COPD disease progression, *P. aeruginosa* has been found to become more prevalent (Beasley et al., 2012).

Respiratory mycobiome also has important links with the respiratory diseases. COPD mycobiome shows diversity and geographic variation. In a study, the effect of fungi in the development of COPD was studied using a sample of patients from three countries (Malaysia, Singapore, and Hong Kong) (Tiew et al., 2020). COPD mycobiome was diverse in stable COPD, and different genera, namely *Alternaria, Cryptococcus, Mycosphaerella*, *Trametes, Aspergillus, Penicillium, Wickerhamomyces*, and *Cladosporium*, were found only in diseased lungs and not in healthy lungs (Martinsen et al., 2021). Also, microbiome enhancement with *Penicillium*, *Curvularia*, and *Aspergillus* species may be associated with a greater mortality rate and higher exacerbation rate, as revealed by clustering analysis. Moreover, a reduction in fungal diversity has been linked to higher mortality rates in COPD patients. Elevated levels of the fungal genera *Penicillium, Trametes, Lodderomyces*, and *Cladosporium* are associated with poor survival rates (Tiew et al., 2021).

### 4.2 Idiopathic pulmonary fibrosis (IPF)

IPF is a persistent, lethal lung parenchymal remodeling disease with an unclear cause (Barratt et al., 2018; Zhang and Wang, 2023). *Haemophilus, Streptococcus, Neisseria*, *Prevotella, Escherichia*, and *Veillonella* are the common bacterial genera that have been identified in the lungs of individuals suffering from IPF (Molyneaux et al., 2014; Fastrès et al., 2017). The population of *Streptococcus* species also increased in IPF patients. Employing culture-independent methods, an elevated bacterial burden in bronchoalveolar lavage fluid, which enables the identification of microbiota specific to the lower lungs of IPF patients, was observed compared to controls (Schneeberger et al., 2019). It is reported that *Firmicutes* dominate in the lower respiratory tract of IPF patients, while *Proteobacteria* is less prevalent. At the genus level, *Streptococcus, Actinomyces, Haemophilus, Neisseria*, and *Veillonella* were reported to be abundant in the lungs of IPF patients (Invernizzi et al., 2021). A correlation between mortality rates in IPF patients and the amount of bacteria in their lungs was established (Molyneaux et al., 2014). Increased levels of granulocyte colony-stimulating factor, IL-1β, IL-1Ra, epidermal growth factors, CXCL8, and macrophage inflammatory protein-1α, as well as aberrant alveolar inflammatory and fibrotic cytokines, are linked to lung microbiota diversity in IPF (O’Dwyer et al., 2019). Furthermore, a positive correlation was found between alveolar concentrations of IL-6 and the relative abundance of the lung *Firmicutes* phylum and a negative relationship between alveolar IL-12p70 and the relative abundance of the lung *Proteobacteria* phylum (O’Dwyer et al., 2019). The fungal composition of IPF lungs has not been studied much and remains a subject that needs to be explored. However, a study has reported higher colonization of *Pneumocystis jiroveci* to be associated with IPF patients (Vidal et al., 2006).

### 4.3 Bronchial asthma

Dysbiosis of the respiratory microbiome is an important factor in the development of asthma. A decrease in the bacteriome diversity is reported in asthma and is associated with the disease severity (Huang et al., 2015; Barcik et al., 2020; Valverde-Molina and García-Marcos, 2023). *Moraxella, Neisseria, Staphylococcus, Actinomyces*, and *Haemophilus* are among the prominent bacterial pathogens associated with asthma exacerbations (Hilty et al., 2010; Mthembu et al., 2021). Other bacteria that have been reported are *Gammaproteobacteria* and *Proteobacteria*, and respiratory commensal bacteria like *Prevotella* and *Veillonella* are found to be less dominant (Hilty et al., 2010). In the lower and upper respiratory tract of children with asthma disease, three bacterial species, *Streptococcus pneumoniae, Moraxella catarrhalis*, and *Haemophilus influenzae*, are predominant (Thorsen et al., 2022). In patients that are hospitalized with atopic asthma, the tracheal microbiome has revealed some prevalent bacteria that feature *Fusobacterium, Sphingimonas, Haemophilus, Porphyromonas*, and *Neisseriaceae*, while *Bacteroides* and *Lactobacillus* were found in low levels (Durack et al., 2017). T-helper cell type 2 (Th2)-high and Th2-low are the two inflammatory endotypes associated with asthma (Wenzel et al., 1999; Kuruvilla et al., 2019). It is reported that fungal diversity is significantly lower in Th2-high individuals than in Th-2-low individuals, and fungal genera associated with type 2 inflammation are *Aspergillus, Cladosporium, Alternaria, Mycosphaerella, Fusarium, Penicillium, Wallemia* and *Trichoderma* (van Woerden et al., 2013). Other fungal genera reported in asthmatic lungs are *Meyerozyma*, *Schizophyllum, Malassezia*, and *Candida* (Chung, 2017). The lung microbiome activates Th2 cells and other pathways and induces chronic inflammation, which may aggravate the progression of asthma. During this inflammation, the growth of some bacterial colonies may be stimulated, and microbial dysbiosis may occur.

### 4.4 Bronchiectasis

Bronchiectasis is a long-term inflammatory lung condition marked by the abnormal dilation of one or more airways (McShane and Tino, 2019; Chen et al., 2024). In these patients, reduced mucociliary function leads to mucus accumulation in certain areas of the airways, which fosters the persistence of fungi and bacteria (Máiz et al., 2015, Máiz et al., 2018). The most prevalent bacterial species identified in bronchiectasis are *P. aeruginosa* and *Haemophilus influenzae*. However, other species have also been identified, including *Staphylococcus aureus, Streptococcus pneumoniae, Moraxella catarrhalis, Escherichia* spp., and *Klebsiella* spp (Chalmers et al., 2014). With a large number of studies being conducted on the respiratory microbiomes, it is now well understood that these diseases are characterized by either dysbiosis (state of the dominance of certain taxa) or loss of diversity of bacterial and fungal species in the microbiome. It is reported that the diseased “dysbiotic” airways are primarily dominated by proteobacteria, such as *Pseudomonas* and *Haemophilus*, which are linked to increased neutrophil-mediated inflammation and exacerbations (Tunney et al., 2013). However, there is a subgroup of patients whose microbiota is dominated by genera *Firmicutes* and *Veillonella* who experience frequent exacerbations despite having lower levels of neutrophilic inflammation (Rogers et al., 2014). A reduction in microbial diversity, particularly marked by a dominance of *Pseudomonas*, was linked to an increased risk of severe bronchiectasis, more frequent episodes of worsening symptoms, and higher mortality rates (Dicker et al., 2021).

**Figure 2:**
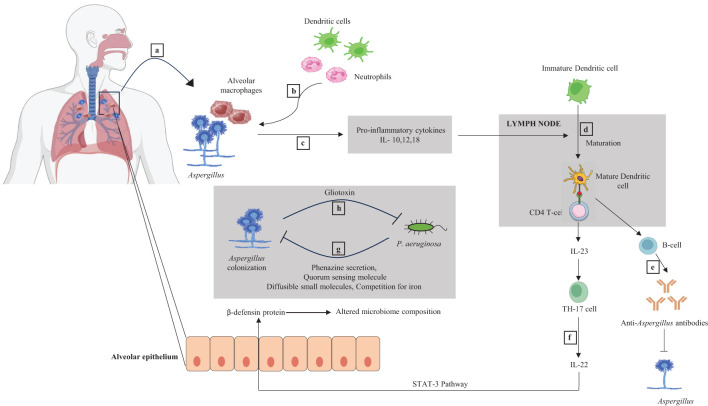
Dynamic interaction between *Aspergillus* and bacteria and their immunological responses. **(a)** Lungs have alveolar macrophage, a component of the innate immune system. Alveolar macrophages recognize the *Aspergillus* species during infection. **(b)** Initial macrophage and fungal interaction recruit more alveolar macrophages, neutrophils, and dendritic cells (DCs) to the infection site. **(c)** Pro-inflammatory cytokines IL-10,12,18 are released as part of the innate immune response, positively stimulating the DCs maturation inside the lymph node. **(d)** Mature DC acts as an antigen-presenting cell (APC) and stimulates naive CD4^+^ T cells. **(e)** CD4^+^ T cells activate the B cell, producing anti-*Aspergillus* antibodies and inhibiting the growth of *Aspergillus*. **(f)** Activated DCs also result in IL-23 secretion from CD4^+^ T cells. IL-23 helps naive CD4^+^ T cells to differentiate into Th-17 cells (a T-cell subtype). Th-17 cells secrete IL-22, which upregulates the expression of an antimicrobial peptide called β-defensin in the alveolar epithelium via the STAT-3 pathway. β-defensin affects the composition of the microbiome in the lungs. **(g)** *P. aeruginosa* may inhibit the colonization of *Aspergillus* by secreting different metabolites like phenazine and quorum sensing molecules (QSMs) and by competing for iron. **(h)** *Aspergillus* can impede the *P. aeruginosa* by release of gliotoxin.

*Aspergillus fumigatus* and *Candida albicans* have been identified as major and common fungus species in the bronchiectasis mycobiome (Mortensen et al., 2011; Chalmers et al., 2014). *Aspergillus* is the principal species distinguishing healthy people from bronchiectasis patients, and its presence has been associated with exacerbations. Thus, *Aspergillus* may have a considerable effect on airway inflammation. A study reported that fungal genera, namely *Cryptococcus, Alternaria, Clavispora, Botrytis*, and *Aspergillus*, were specifically identified only in bronchiectasis patients and not in the healthy controls. However*, Saccharomyces, Candida*, and *Penicillium* were reported in both bronchiectasis patients and healthy controls (Aogáin et al., 2018). *Aspergillus* spp., can strongly induce immunological responses such as interleukin-22 at the level of the respiratory mucosa. This, in turn, controls the production of defensins, which are peptides with antimicrobial action that affect the makeup of the pulmonary bacterial microbiome, which includes *P. aeruginosa. Aspergillus* spp., also cause an increase in lung macrophages and a Th2 and Th17 response (Kim et al., 2014; Baker and Dickson, 2025) (**Figure 2**).

We provided an overview of the human respiratory microbiome and the ways in which the microbiome of a healthy person varies from that of people with different respiratory conditions in the first part. It makes more sense to research the human pulmonary microbiome along with techniques used to identify the bacteria species. As a result, we have also attempted to provide an overview of the techniques that have enabled the thorough investigation of the human microbiome in the following section. Different respiratory diseases have distinct microbial profiles, with certain species being more numerous in one while being missing or less prevalent in another. These techniques have aided in the identification of microbial interactions and the classification of both pathogenic and commensal species in the human microbiome. An overview of the techniques employed in the investigation of the human microbiome, including some that have received recent attention, is given in this literature. The human microbiome is comprehensively investigated using the “meta-omics” technique. Among these are metagenomics, which examines genes, metatranscriptomics, which analyzes transcripts, metaproteomics, which studies the proteome, metabolomics, which analyzes metabolites produced by the microbiome, and 16S rRNA gene sequencing, which determines the phylogenetic marker in bacteria and the internal transcribed spacer (ITS) region in fungi (Takeuchi et al., 2025a). Another new and effective technique is single cell genomics, which is mostly used to examine the low abundance of microorganisms in a microbiome sample and also aids in examining the heterogeneity even within a single strain. The relevant section contains a detailed discussion of the procedures.

## 5 Methods to study human microbiome

Currently, several methods are available to study the human microbiome, and in this review, we have tried to emphasize how these methods have been used to study different aspects of the human microbiome along with advantages and disadvantages of these methods (**Tables 1**, **2**).

**Table 1:** Methods utilized to study the human microbiome.

Method of study	Microbiome studied	Purpose of the study	Results	References
1. GENE MARKER ANALYSIS				
Sequencing of 16S rRNA	Respiratory microbiome	To study the characteristics of sputum microbiota in patients having acute exacerbation of other disease states of COPD	Bacterial diversity was decreased in patients having acute exacerbated COPD and bacterial composition differed from patients with stable COPD and healthy controls	(Chang et al., 2021)
	Respiratory microbiome	To study the bacterial profile of respiratory microbiome in healthy smokers and non-smokers	The relative abundance of *Neisseria, Gemela*, and *Porphyromonas* was reduced in smokers.	(Long et al., 2020)
	Gut microbiome	To study the profound effect of ciprofloxacin antibiotic on gut microbiome of human	Approximately 30% of the gut’s taxa experienced relative abundance changes due to Ciprofloxacin treatment.	(Jiang et al., 2022)
Sequencing of Internal Transcribed Spacer (ITS) region	Respiratory microbiome	To study aberrant mycobiome in the respiratory tract after lung transport	The subjects with lung transplants showed differences in the microbial load and community composition and had reduced fungal diversity when compared to healthy control groups.	(Wilmanski et al., 2019a)
	Gut microbiome	To characterize the gut fungal microbiome (mycobiome) of the Human Microbiome Project (HMP) cohort; ITS2 region sequencing.	Results demonstrated that gut mycobiome is less diverse than gut bacteriome and *Saccharomyces, Malassezia*, and *Candida* are the most prevalent yeast genera in this cohort.	(Vidal et al., 2006)
2. SINGLE CELL GENOMICS	Human microbiome (skin and gut microbiota)	To reconstruct strain-specific genomes from the human microbiome using a combined approach of single-cell genomics and metagenomics.	A high-quality draft genome was produced from both mock and human microbiome samples using single-cell metagenomics method genes.	(Arikawa et al., 2021)
	Oral microbiome	Isolation and amplification of a rare and uncultivated bacterial cell (TM7 phylum; cells having rod-like morphotype) from oral microbiota of human and sequenced their DNA gain genetic insights.	Individual TM7 cells’ genome amplification allowed sequencing and assembly of more than 1,000 genes, and the physiology of members of this phylum was studied.	(Marcy et al., 2007)
3. SHOTGUN METAGENOMICS	Gastric microbiome	To assess the role and makeup of the gastric microbiota in advanced gastric adenocarcinoma (GC) as well as in superficial gastritis (SG).	The most prevalent taxa identified in GC are commensals or opportunistic pathogens also present in the oral cavity. Pathways for lipopolysaccharide (LPS) and L-arginine biosynthesis were enhanced in GC, while those for short-chain fatty acid (SCFA) fermentation and branched-chain amino acid metabolism were more enriched in SG.	(Hu et al., 2018)
	Gut microbiome	Profiling of human gut microbiome in COVID-19 patients to study change caused during the disease	Four bacterial species were identified to be unique to COVID-19 patients, namely *Prevotella bivia*, *Streptococcus thermophilus*, *Fusobacterium ulcerans* and *Bacteroides oleiciplenus*	(Li et al., 2021)
4. METATRANSCRIPT -OMICS	Oral microbiome	Metatranscriptomic analysis of the human oral microbiome in both healthy and diseased states.	Microbiota associated with periodontal disease are more similar to each other than those linked to healthy states. Average microbial composition for both healthy and disease-related plaque was identified. The metabolic gene expression of different species showed significant variability among patients.	(Jorth et al., 2014)
	Lung microbiome	To study the metatranscriptome associated with human lung cancer.	Fifty taxonomic groups were identified, out of that seven taxonomic groups were linked to lung cancer patient’s survival. Cytotoxic T-cells, naive T helper cells, dendritic cells, and central memory T helper cells were linked to improved overall survival, whereas pro-B-cell enhancement indicated poorer survival outcomes.	(Chang et al., 2021)
5. METAPROTEOMICS	Gut microbiome	To investigate the functional roles of the human gut microbiome in colorectal cancer by using metaproteomics	Healthy individuals had 17% more identified proteins than colorectal cancer (CRC) patients, indicating that CRC patients exhibit lower microbial protein diversity. Taxonomic abundance differences were observed in the gut microbiome of colorectal cancer patients compared to healthy controls, with *Methanobacteriaceae, Sporolactobacillaceae*, and *Desulfobacterales* being abundant in the CRC patients	(Long et al., 2020)
	Oral microbiome	To perform a detailed metaproteomics analysis of the oral microbiome to identify specific protein profiles associated with lung cancer	Compared to the conventional metaproteomics approach, interfering peptides decreased by 52.87%, while bacterial peptides and species increased by 94.97% and 44.90%, respectively. The decrease in the interfering peptide increased the reproducibility.	(Jiang et al., 2022)
METABOLOMICS	Gut microbiome	To study alpha diversity of the human gut microbiome using blood metabolome	Shannon diversity predictions effectively represented shifts in gut microbial diversity related to gastrointestinal health, underscoring a strong connection between the structure of the gut microbiome and host physiology.	(Wilmanski et al., 2019a)

**Table 2:** Pros and cons of the methods used to study human microbiome.

Method of study	Pros	Cons	References
1. GENE MARKER ANALYSIS	Cost-effective and gives insight into microbiome diversity and abundance.	Less information is revealed for low abundant members and lacks functional insight, PCR can result in a biased abundance composition. ITS sequences lack a well-established database.	(Nilsson et al., 2006; Kembel et al., 2012; Walker et al., 2015)
2. SINGLE-CELL GENOMICS	Useful for the study of microdiversity within a single strain and low abundant members of the microbiome.	Aggregation of bacterial cells, bacterial cell wall challenges sequencing of single cells.	(Elowitz et al., 2002; Hatzenpichler et al., 2020; Bowers et al., 2022)
3. SHOTGUN METAGENOMICS	All microbial genomes can be captured, allowing the study of different taxa, their abundance, and gene coding sequences.	Difficult to determine the genome from where a particular read has originated, high sequencing cost.	(Xia et al., 2011; Sharpton, 2014; Quince et al., 2017);
4. METATRANSCRIPTOMICS	Identify the transcriptionally active organism’s taxonomy and the function of their expressed genes.	Requires high-quality RNA, fails to sense short-lived response generated against stimuli as mRNA has a short half-life, and requires an integrative omics approach as mRNA presence may not imply the presence of protein.	(Bashiardes et al., 2016)
5. METAPROTEOMICS	Determine microbiome composition, physiology, and metabolism of each individual microorganism, decipher the host-microbiome interactions	Lack of databases, bioinformatics tools, poor yield of proteins, poor peptide identification.	(Kleiner, 2019; Wang et al., 2020; Salvato et al., 2021)
6. METABOLOMICS	Profiles of metabolites produced within a microbiome help in studying host-microbiome interaction. Can detect both endogenous and exogenous substance	Origin of the metabolite is not known	(Lamichhane et al., 2018; Zierer et al., 2018)

### 5.1 Gene marker analysis

Targeted sequencing is the approach most frequently employed to examine the microbiome. Certain genes are sequenced, including the 16S ribosomal RNA (rRNA) gene for bacteria and the internal transcribed spacer (ITS) region for fungi. Determining and recognizing a unique sequence is one of the challenges in marker gene investigations present (Galloway-Peña and Hanson, 2020; Pei et al., 2023). Hypervariable areas in both the ITS and 16S rRNA genes can alter quickly and even vary within a single cell (Yang et al., 2016). Additionally, it can be difficult to distinguish between different taxa and define a unique sequence when they share similar gene sequences, as in the case of *Escherichia* and *Shigella* (Větrovský and Baldrian, 2013). Therefore, a method of binning sequences called operational taxonomic units (OTUs) is now being used. The 16S rRNA gene is amplified and sequenced to create the most common type of comprehensive microbiome taxonomic data, and the acquired sequences are typically clustered into OTUs based on the overall percent similarity of the sequences. The relative abundance of each OTU in the sample is then determined (Ju and Zhang, 2015). Each OTU’s taxonomy can be deduced by grouping the reads with reference sequences of known taxonomy or by using a classification algorithm. This algorithm estimates the probable taxonomy of each OTU or individual read (Ju and Zhang, 2015; Kopylova et al., 2016).

Although the targeted amplicon technique only provides information about bacteria within a community, it can also be adapted to explore fungal species. Amplification of the fungal ITS regions is the most popular method. The two ITS regions called ITS1 and ITS2, are situated between the 18S and 5.8S rDNA and 5.8S and 26S rDNA genes, respectively (Schoch et al., 2012). The genetic locus containing the regions ITS1 and ITS2, which encode for the nonfunctional RNA transcribed during rRNA synthesis, as well as the 18S, 5.8S, and 28S rRNA genes, have been the main targets for culture-independent analyses and database generation (De Filippis et al., 2017). The utilization of pyrosequencing in NGS techniques, along with the construction of clone libraries from the 18S, 5.8S, and 28S regions, along with ITS1 and ITS2, have unveiled a pool of undisclosed variety of fungi in the microbiome, both culturable and nonculturable (Siqueira et al., 2012; Akaçin et al., 2022). However, the two most common problems faced in ITS sequencing are: first, in contrast to bacterial 16S sequences, ITS sequences lack a well-established database. Second, fungal ITS sequences from various species can vary greatly in size and sequence content (Abarenkov et al., 2010). Also, PCR amplification can result in biased abundance comparisons if the ribosomal genes of some taxa are poorly amplified (Walker et al., 2015).

In a study by Morris et al. (2013), gene marker analysis method was applied to study and compare the respiratory microbiome of the healthy non-smokers and smokers. Bacterial species like *Methylobacterium, Enterobacteriaceae, Ralstonia* and *Haemophilus* were significantly reduced in the lung microbiome compared to the value predicted by the neutral model (Du et al., 2024). The lung bacterial population of smokers and non-smokers was found to be same, however, the mouth bacterial community differed in species such as *Porphyromonas, Gemella* and *Neisseria*. Thus, the gene marker analysis method helped to reveal that smoking does not significantly alter the lung microbiome (Morris et al., 2013). Charlson et al., 2012 also used gene marker analysis method to study the abberant fungal and bacterial microbiome of lung transplant recepients. Using the fungal internal transcribed spacer and bacterial 16S rDNA sequencing, the bacterial and fungal species were profiled. URT was sampled by oropharyngeal wash and lung by Bronchoalveolar lavage (BAL). A high bacterial burden was observed in the BAL in lung transplant recipients as compared to the control group. Fungal population in BAL was dominated by *Aspergillus*, while *Candida* was found to be dominant at both the sites (OW and BAL) (Charlson et al., 2012).

### 5.2 Single-cell genomics

The single-cell genomics technique involves isolating a single microbial cell from a heterogenous microbiome sample and sequencing each individually isolated cell of a microbiome to obtain the information. This technique is helpful in studying low abundant microorganisms present in a microbiome sample, which are not covered well by common sequencing methods like 16S rRNA gene amplification and sequencing (Hatzenpichler et al., 2020; Bowers et al., 2022). Microbial cell populations can show heterogeneity in gene expression; thus, they can be heterogeneous even within a single strain; this type of microdiversity can be studied using a single-cell genomics approach (Elowitz et al., 2002). However, isolating single microbial cells from a heterogenous sample is difficult as bacteria often show aggregation (Nwoko and Okeke, 2021). Moreover, the bacterial cell wall is required to be permeabilized as it poses difficulty in sequencing single cells (Blattman et al., 2020). Single cells are isolated using techniques like fluorescence-activated cell sorting (FACS), micromanipulation, and microfluidics (Tolonen and Xavier, 2017). FACS is a high throughput technique that can isolate single cells based on their size, internal complexity, and fluorescence. Micromanipulation involves the use of micropipettes and an inverted microscope, and single cells are isolated from a heterogeneous mixture. It is a time-consuming and labor-intensive method (Blainey, 2013). Microfluidics offers high throughput isolation of single cells and individual genome barcoding (Leung et al., 2012). Unlike metagenomics, single-cell genomics does not depend on the clonality of microbial populations; instead, it retrieves genome sequences from individual cells. Amplification biases may occur during DNA amplification, which results in incomplete genome sequences. Therefore, the single-cell genomics technique is highly accurate when the single-cell sequencing data are co-assembled as it minimizes the gaps and errors that may present in the individual single-cell amplified genomes (Kogawa et al., 2018).

### 5.3 Shotgun metagenomics method

It is a powerful non-culture-based technique. In shotgun metagenomics, all of the microbial genomes in a sample are captured by means of untargeted sequencing techniques (Quince et al., 2017; Sharpton, 2014). Depending on the sequencing depth, it is possible to identify every DNA genome that is present in a sample, and since complete genetic repertoire is extracted, it can determine different taxa that are present in the sample with their relative abundance. It can also decipher information about the gene coding sequences (Qin et al., 2010; Xia et al., 2011). In this method, DNA is extracted from all cells in the community and broken into small fragments rather than targeted and amplified in a specific genomic region. These fragments are then sequenced separately. This produces DNA sequences, or “reads,” corresponding to various genomic regions from the many organisms present in the sample, including non-microbial species. Some of the sequences will come from coding regions that reveal the biological functions encoded in the genomes, while others come from taxonomically important regions like 16S rRNA (Sharpton, 2014). Like any other technique, shotgun metagenomics also has certain disadvantages. This technique presents complex and large data; the analysis process is difficult, and it is often difficult to determine the genome from where a particular read originated. Additionally, while studying the microbiome, metagenomes must be separated from contaminating host DNA (Chew and Holmes, 2009). The adaptability of this approach is restricted by the high expense of sequencing each sample, in addition to the technical difficulties of DNA shearing, library creation, and bioinformatics pipelines (Usyk et al., 2023). Also, finding and eliminating contaminants from metagenomic sequences is particularly challenging, and it is hard to assess which read has resulted from a contaminant metagenome. If a metagenomic contaminant’s genome is enriched for rare genes, it can skew assessments of community genetic diversity. This is particularly true if the contaminant is very prevalent or has a big genome (Schmieder and Edwards, 2011).

### 5.4 Metatranscriptomics

Metatranscriptomics can be applied to study the gene expression of the bacterial communities present at a site within or on the human host. Gene expression profiling can be done for both culturable and non-culturable bacteria. The data of metatranscriptomic analysis complement data obtained in metagenomics analysis since this helps to precisely identify which genes are transcribed out of all the genes that were annotated in the metagenomics analysis. It elucidates the extent to which the genes are transcribed and, therefore, helps in determining the function of these genes (Franzosa et al., 2014). For metatranscriptomic analysis of the microbiome, total RNA is isolated from the bacterial community colonizing a particular niche. Only up to 5% of all RNA species are prokaryotic mRNA, the majority being the 16S and 23S rRNAs and tRNAs (Peano et al., 2013). Eukaryotic mRNAs contain a poly-A tail, which can be used to obtain cDNA by using oligo dT primers, while prokaryotic mRNAs lack a poly-A tail. Thus, their isolation is not simple. Prokaryotic mRNAs can be isolated using probes attached to a magnetic bead. They anneal to their complementary target sequences, and magnetic beads help in their isolation (Sultan et al., 2014). RNase inhibitors are added during the extraction process to prevent their degradation from ribonucleases. The RNA obtained is cut into smaller fragments, and cDNA is made using oligo dT primers. Adaptors are ligated to cDNA ends, generating a representative library that is subjected to amplification and sequencing (Bikel et al., 2015). Illumina sequencing methodologies are commonly incorporated in shotgun metagenomics and metatranscriptomics as it is a high throughput technique and comparatively cheaper (Galloway-Peña and Hanson, 2020). The sequenced mRNAs are called RNA sequence reads, and to identify the transcriptionally active organism’s taxonomy and the function of their expressed genes, these RNA sequence reads are mapped to different genomes and pathways (Galloway-Peña and Hanson, 2020). Metatranscriptomics data obtained from a microbiome sample can be aligned and assembled using bioinformatics tools like SOAPdenovo (Xie et al., 2014). Certain factors that limit the use of metatranscriptomics include the requirement of high-quality RNA from the microbiome sample. mRNA has a short half-life and is prone to degradation by nucleases; therefore, sometimes, it fails to sense the responses generated against stimuli. Moreover, the presence of mRNA may not always imply the presence of protein, and an integrative omics approach is required to study the microbiome sample better (Bashiardes et al., 2016). In a study by Chang et al. (2021), metatranscriptomics method was employed to analyse the human lung microbiome in patients with lung cancer. RNA-Seq was performed for the investigation of the human lung cancer metatranscriptome. The study revealed that a number of bacterial species that significantly associated with the prognosis were enriched in the lung cancer tissues (Emadi et al., 2025a). It included *Staphylococcus* spp, *B. diminuta, M. franklinii, E. cloacae, M. chelonae, A. radioresistens, B. megaterium, R. erythropolis* and *P. aeruginosa.*

### 5.5 Metaproteomics

Metaproteomics deals with studying proteins expressed by a microbiome sample. It can also be used to study the proteins expressed within any microbiome community at a given time (Wilmes and Bond, 2004). However, to correctly identify the peptide and to analyze pathways, metaproteomics requires databases and libraries created using metagenomics (Lai et al., 2019). It is also helpful in determining the composition of the microbiome, physiology, and metabolism of each microorganism that is part of the microbiome. Using metaproteomics, the host gene expression and the microbial gene expression can be studied, and thus, it can help decipher the host-microbiome interactions that may be involved in causing disease (Salvato et al., 2021). Metaproteomics has not been used as much as metatranscriptomics and metabolomics because of a lack of databases, bioinformatics tools, poor yield of proteins, and poor peptide identification (Kleiner, 2019; Wang et al., 2020). It takes access to both an enriched protein sequence database and instrumentations like high-resolution liquid chromatography (HRLC) and mass spectrometry to perform metaproteomics of a sample. A protein database predicts a theoretical spectrum for a sample to which the experimentally obtained mass spectra are compared, provided that the database has the expected protein sequence. A successful metaproteomic study results in good protein extraction efficiency, separation efficiency, and unambiguous identification (Hettich et al., 2013; Tanca et al., 2016; Rechenberger et al., 2019).

### 5.6 Metabolomics

Metabolomics analyzes the metabolites produced by the microbiota and studies the interaction of these metabolites with both the host metabolism and the microbiota (Lamichhane et al., 2018). Bacteria produce small molecules as part of their metabolic activity; for example, *P. aeruginosa* secretes different metabolites like phenazine and quorum sensing molecules, which may inhibit the colonization of *Aspergillus.* Host antifungal immunity can be modulated by molecules released by microbiota during their metabolic activity. *P. aeruginosa* can produce metabolites in the CF lung that significantly affect how it interacts with *A. fumigatus.* Recently, a study of metabolomics using molecular networking showed that xenobiotics, specialized metabolites from *P. aeruginosa*, and host sphingolipids make up the chemical composition of CF sputa (Quinn et al., 2016). However, the microbial metabolites were not the same as those produced by laboratory cultures, and the *P. aeruginosa*-derived quinolone signal was not present in sputum, even in the presence of its precursor molecules, but was easily identified from cultured strains. This reflects that the signals provided by the CF lung chemical environment affect the *P. aeruginosa* metabolism (Mowat et al., 2010; Quinn et al., 2016).

These metabolites can be detected using metabolomics, and techniques like mass spectrometry (MS) are often used to identify known metabolites (Zierer et al., 2018). A complex biological sample contains diverse metabolites that can be sensed and quantified using mass spectrometry because of its high sensitivity (Aksenov et al., 2017). Tools used for metabolomic studies can detect both endogenous and exogenous substances. MS can be used for the detection and quantification of metabolites released by the microbiome with the aid of different samples, including solids (involves detecting the metabolites directly from the surface) or volatiles (microbial volatile organic compounds released by the microbiome) (Shih et al., 2014; Weisskopf et al., 2021). Biotyping is a common strategy to identify microbial metabolites from a clinical sample. It utilizes the Matrix-assisted laser desorption/ionization-Time of flight (MALDI-TOF) technique to profile the ribosomal proteins present in bacteria colonies, and the data obtained is compared with the MS pattern obtained from a clinically important microorganism that has been well characterized. This helps in the taxon identification of the sample (Freiwald and Sauer, 2009). MS Imaging (MSI) has also been used to study the metabolic exchange between two or even more microorganisms (Watrous et al., 2013).

## 6 Conclusion

It has only just been apparent how the respiratory microbiomes contribute to colonization resistance and immune system activation despite long being overlooked. However, to completely understand the human respiratory microbiome ecosystem’s relationship to human physiology and illness, it is necessary to understand its mechanism of interaction. An important part of this microbial ecology comes from the oral-lung and gut-lung axis (Bongers et al., 2024). A disturbed respiratory microbiota is a hallmark of four chronic respiratory diseases: asthma, COPD, CF, and IPF. Each of these illnesses has been found to have unique mucosal microbial signatures. Pulmonary medicine might be revolutionized by identifying small molecule metabolites originating from the microbiome and how they affect the host’s inflammatory and immunological responses. Incorporating the respiratory microbiome into pathophysiologic concepts of chronic disease will further our understanding of lung respiratory microbiomes and the development of new therapeutic approaches.

## References

[B1] Abarenkov K. Nilsson R. H. Larsson K. H. Alexander I. J. Eberhardt U. Erland S.. (2010). The UNITE database for molecular identification of fungi–recent updates and future perspectives. New Phytol. 186, 281–285. doi: 10.1111/j.1469-8137.2009.03160.x 20409185

[B2] Akaçin İ. Ersoy Ş. Doluca O. Güngörmüşler M. (2022). Comparing the significance of the utilization of next generation and third generation sequencing technologies in microbial metagenomics. Microbiological Res. 264. doi: 10.1016/j.micres.2022.127154 35961096

[B3] Aksenov A. A. Da Silva R. Knight R. Lopes N. P. Dorrestein P. C. (2017). Global chemical analysis of biology by mass spectrometry. Nat. Rev. Chem. 1. doi: 10.1038/s41570-017-0054

[B4] Angebault C. Vandenborght L.-E. Bassinet L. Wizla N. Ferroni A. Dessein R.. (2025). Airway mycobiota—Microbiota during pulmonary exacerbation of cystic fibrosis patients: A culture and targeted sequencing study. Mycoses 68, e70024. doi: 10.1111/myc.70024 39816006 PMC11736540

[B5] Aogáin M. Mac Chandrasekaran R. Hou Lim A. Y. Low T. B. Tan G. L. Hassan T.. (2018). Immunological corollary of the pulmonary mycobiome in bronchiectasis: The CAMEB study. Eur. Respir. J. 52. doi: 10.1183/13993003.00766-2018 PMC6092680 29880655

[B6] Arikawa K. Ide K. Kogawa M. Saeki T. Yoda T. Endoh T.. (2021). Recovery of strain-resolved genomes from human microbiome through an integration framework of single-cell genomics and metagenomics. Microbiome 9. doi: 10.1186/s40168-021-01152-4 PMC8507239 34641955

[B7] Baker & Dickson (2025). The microbiome and pulmonary immune function. Clinics Chest Med. 46, 77–91. doi: 10.1016/j.ccm.2024.10.006 39890294

[B8] Bakhti S. Z. Latifi-Navid S. (2021). ). Oral microbiota and Helicobacter pylori in gastric carcinogenesis: what do we know and where next? BMC Microbiol. 21. doi: 10.1186/s12866-021-02130-4 PMC7934379 33663382

[B9] Barcik W. Boutin R. C. T. Sokolowska M. Finlay B. B. (2020). The role of lung and gut microbiota in the pathology of asthma. Immunity 52, 241–255. doi: 10.1016/j.immuni.2020.01.007 32075727 PMC7128389

[B10] Barratt S. L. Creamer A. Hayton C. Chaudhuri N. (2018). Idiopathic pulmonary fibrosis (IPF): An overview. J. Clin. Med. 7. doi: 10.3390/jcm7080201 PMC6111543 30082599

[B11] Bashiardes S. Zilberman-Schapira G. Elinav E. (2016). Use of metatranscriptomics in microbiome research. Bioinf. Biol. Insights 10, 19–25. https://doi.org/doi: 10.4137/BBI.S34610 PMC4839964 27127406

[B12] Beasley V. Joshi P. V. Singanayagam A. Molyneaux P. L. Johnston S. L. Mallia P. (2012). Lung microbiology and exacerbations in COPD. Int. J. COPD 7, 555–569. doi: 10.2147/COPD.S28286 PMC3437812 22969296

[B13] Belizário J. Garay-Malpartida M. Faintuch J. (2023). Lung microbiome and origins of the respiratory diseases. Curr. Res. Immunol. 4. doi: 10.1016/j.crimmu.2023.100065 PMC10339129 37456520

[B14] Belkaid Y. Hand T. W. (2014). Role of the microbiota in immunity and inflammation. Cell 157, 121–141. doi: 10.1016/j.cell.2014.03.011 24679531 PMC4056765

[B15] Bikel S. Valdez-Lara A. Cornejo-Granados F. Rico K. Canizales-Quinteros S. Soberón X.. (2015). Combining metagenomics, metatranscriptomics and viromics to explore novel microbial interactions: Towards a systems-level understanding of human microbiome. Comput. Struct. Biotechnol. J. 13, 390–401. doi: 10.1016/j.csbj.2015.06.001 26137199 PMC4484546

[B16] Blainey P. C. (2013). The future is now: Single-cell genomics of bacteria and archaea. FEMS Microbiol. Rev. 37, 407–427. doi: 10.1111/1574-6976.12015 23298390 PMC3878092

[B17] Blattman S. B. Jiang W. Oikonomou P. Tavazoie S. (2020). Prokaryotic single-cell RNA sequencing by in *situ* combinatorial indexing. Nat. Microbiol. 5, 1192–1201. doi: 10.1038/s41564-020-0729-6 32451472 PMC8330242

[B18] Bongers K. S. Massett A. O‘wyer D. N. (2024). The oral–lung microbiome axis in connective tissue disease-related interstitial lung disease. Semin. Respir. Crit. Care Med. 45, 449–458. doi: 10.1055/s-0044-1785673 38626906

[B19] Bosch A. A. Levin E. van Houten M. A. Hasrat R. Kalkman G. Biesbroek G.. (2016). Development of upper respiratory tract microbiota in infancy is affected by mode of delivery. EBioMedicine 9, 336–345. doi: 10.1016/j.ebiom.2016.05.031 27333043 PMC4972531

[B20] Bowers R. M. Nayfach S. Schulz F. Jungbluth S. P. Ruhl I. A. Sheremet A.. (2022). Dissecting the dominant hot spring microbial populations based on community-wide sampling at single-cell genomic resolution. ISME J. 16, 1337–1347. doi: 10.1038/s41396-021-01178-4 34969995 PMC9039060

[B21] Bresser P. Out T. A. Van Alphen L. Jansen H. M. Lutter R. (2000). Airway inflammation in nonobstructive and obstructive chronic bronchitis with chronic Haemophilus influenzae airway infection: Comparison with noninfected patients with chronic obstructive pulmonary disease. Am. J. Respir. Crit. Care Med. 162, 947–952. doi: 10.1164/ajrccm.162.3.9908103 10988111

[B22] Budden K. F. Romani L. (2025). Editorial: Lung microbiome in health and disease. Front. Pharmacol. 16. doi: 10.3389/fphar.2025.1565849 PMC11869607 40028156

[B23] Campbell C. D. Gleeson M. Sulaiman I. (2023). The role of the respiratory microbiome in asthma. Front. Allergy 4. doi: 10.3389/falgy.2023.1120999 PMC10262749 37324782

[B24] Chalmers J. D. Goeminne P. Aliberti S. McDonnell M. J. Lonni S. Davidson J.. (2014). The bronchiectasis severity index an international derivation and validation study. Am. J. Respir. Crit. Care Med. 189, 576–585. doi: 10.1164/rccm.201309-1575OC 24328736 PMC3977711

[B25] Chang Y. S. Hsu M. H. Tu S. J. Yen J. C. Lee Y. T. Fang H. Y.. (2021). Metatranscriptomic analysis of human lung metagenomes from patients with lung cancer. Genes 12. doi: 10.3390/genes12091458 PMC8466605 34573440

[B26] Charlson E. S. Diamond J. M. Bittinger K. Fitzgerald A. S. Yadav A. Haas A. R.. (2012). Lung-enriched organisms and aberrant bacterial and fungal respiratory microbiota after lung transplant. Am. J. Respir. Crit. Care Med. 186, 536–545. doi: 10.1164/rccm.201204-0693OC 22798321 PMC3480531

[B27] Chen C.-M. Hou H.-H. Chien N. Lu K.-Z. Lin C.-H. Liao Y.-C.. (2024). The clinical impacts of lung microbiome in bronchiectasis with fixed airflow obstruction: a prospective cohort study. Respir. Res. 25, 308. doi: 10.1186/s12931-024-02931-x 39143556 PMC11325704

[B28] Chew Y. V. Holmes A. J. (2009). Suppression subtractive hybridisation allows selective sampling of metagenomic subsets of interest. J. Microbiological Methods 78, 136–143. doi: 10.1016/j.mimet.2009.05.003 19442689

[B29] Chung K. F. (2017). Airway microbial dysbiosis in asthmatic patients: A target for prevention and treatment? J. Allergy Clin. Immunol. 139, 1071–1081. doi: 10.1016/j.jaci.2017.02.004 28390574

[B30] Cicinelli E. Ballini A. Marinaccio M. Poliseno A. Coscia M. F. Monno R.. (2012). Microbiological findings in endometrial specimen: our experience. Arch. gynecology obstetrics 285, 1325–1329. doi: 10.1007/s00404-011-2138-9 22113463

[B31] Cuerpo S. Moisés J. Hernández-González F. Benegas M. Ramirez J. Sánchez M.. (2019). Acute exacerbations of idiopathic pulmonary fibrosis: Does clinical stratification or steroid treatment matter? Chronic Respir. Dis. 16. doi: 10.1177/1479973119869334 PMC6704413 31431063

[B32] Cui L. Morris A. Ghedin E. (2013). The human mycobiome in health and disease. Genome Med. 5. doi: 10.1186/gm467 PMC3978422 23899327

[B33] de Dios Caballero J. Cantón R. Ponce-Alonso M. García-Clemente M. M. Gómez G. de la Pedrosa E. López-Campos J. L.. (2022). The human mycobiome in chronic respiratory diseases: current situation and future perspectives. Microorganisms 10. doi: 10.3390/microorganisms10040810 PMC9029612 35456861

[B34] De Filippis F. Laiola M. Blaiotta G. Ercolini D. (2017). Different amplicon targets for sequencing-based studies of fungal diversity. Appl. Environ. Microbiol. 83. doi: 10.1128/AEM.00905-17 PMC5561290 28625991

[B35] Dicker A. J. Lonergan M. Keir H. R. Smith A. H. Pollock J. Finch S.. (2021). The sputum microbiome and clinical outcomes in patients with bronchiectasis: a prospective observational study. Lancet Respir. Med. 9, 886–895. doi: 10.1016/S2213-2600(20)30557-9 33961805

[B36] Dickson R. P. Erb-Downward J. R. Freeman C. M. McCloskey L. Beck J. M. Huffnagle G. B.. (2015). Spatial variation in the healthy human lung microbiome and the adapted island model of lung biogeography. Ann. Am. Thorac. Soc. 12, 821–823. doi: 10.1513/AnnalsATS.201501-029OC 25803243 PMC4590020

[B37] Dickson R. P. Erb-Downward J. R. Freeman C. M. McCloskey L. Falkowski N. R. Huffnagle G. B.. (2017). Bacterial topography of the healthy human lower respiratory tract. MBio 8, 245–257. doi: 10.1128/mBio.02287-16 PMC5312084 28196961

[B38] Dickson R. P. Erb-Downward J. R. Huffnagle G. B. (2013). The role of the bacterial microbiome in lung disease. Expert Rev. Respir. Med. 7. doi: 10.1586/ers.13.24 PMC4007100 23734647

[B39] Du C. Zhang Y. Zhang H. Zhang H. Liu J. Shen N. (2024). Bibliometric analysis of research trends and prospective directions of lung microbiome. Pathogens 13, 996. doi: 10.3390/pathogens13110996 39599549 PMC11597221

[B40] Durack J. Lynch S. V. Nariya S. Bhakta N. R. Beigelman A. Castro M.. (2017). Features of the bronchial bacterial microbiome associated with atopy, asthma, and responsiveness to inhaled corticosteroid treatment. J. Allergy Clin. Immunol. 140,, 63–75. doi: 10.1016/j.jaci.2016.08.055 27838347 PMC5502827

[B41] Eidi S. Kamali S. A. Hajari Z. Fata A. Farid Hosseini R. Naseri A.. (2016). Nasal and indoors fungal contamination in healthy subjects. Health Scope 5. doi: 10.17795/jhealthscope-30033

[B42] Elowitz M. B. Levine A. J. Siggia E. D. Swain P. S. (2002). Stochastic gene expression in a single cell. Science 297, 1183–1186. doi: 10.1126/science.1070919 12183631

[B43] Emadi. (2024a).Impact of COVID-19 on gut–lung microbiota a mini review (Accessed December 27, 2024).

[B44] Emadi R. Saki S. Yousefi P. Tabibzadeh A. (2025a). A perspective on lung cancer and lung microbiome: insight on immunity. Immunity Inflammation Dis. 13, e70145. doi: 10.1002/iid3.70145 PMC11783403 39887959

[B45] Fastrès A. Felice F. Roels E. Moermans C. Corhay J. L. Bureau F.. (2017). The lung microbiome in idiopathic pulmonary fibrosis: A promising approach for targeted therapies. Int. J. Mol. Sci. 18. doi: 10.3390/ijms18122735 PMC5751336 29258183

[B46] Franzosa E. A. Morgan X. C. Segata N. Waldron L. Reyes J. Earl A. M.. (2014). Relating the metatranscriptome and metagenome of the human gut. Proc. Natl. Acad. Sci. United States America 111. doi: 10.1073/pnas.1319284111 PMC4050606 24843156

[B47] Freiwald A. Sauer S. (2009). Phylogenetic classification and identification of bacteria by mass spectrometry. Nat. Protoc. 4, 732–742. doi: 10.1038/nprot.2009.37 19390529

[B48] Gallacher D. J. Kotecha S. (2016). Respiratory microbiome of new-born infants. Front. Pediatr. 4. doi: 10.3389/fped.2016.00010 PMC4762994 26942168

[B49] Galloway-Peña J. Hanson B. (2020). Tools for analysis of the microbiome. Digestive Dis. Sci. 65, 674–685. doi: 10.1007/s10620-020-06091-y PMC7598837 32002757

[B50] Garcia-Nuñez M. Millares L. Pomares X. Ferrari R. Pérez-Brocal V. Gallego M.. (2014). Severity-related changes of bronchial microbiome in chronic obstructive pulmonary disease. J. Clin. Microbiol. 52, 4217–4223. doi: 10.1128/JCM.01967-14 25253795 PMC4313290

[B51] Hatzenpichler R. Krukenberg V. Spietz R. L. Jay Z. J. (2020). Next-generation physiology approaches to study microbiome function at single cell level. Nat. Rev. Microbiol. 18, 241–256. doi: 10.1038/s41579-020-0323-1 32055027 PMC7133793

[B52] Hettich R. L. Pan C. Chourey K. Giannone R. J. (2013). Metaproteomics: Harnessing the power of high performance mass spectrometry to identify the suite of proteins that control metabolic activities in microbial communities. Analytical Chem. 85, 4203–4214. doi: 10.1021/ac303053e PMC3696428 23469896

[B53] Hilty M. Burke C. Pedro H. Cardenas P. Bush A. Bossley C.. (2010). Disordered microbial communities in asthmatic airways. . PloS One 5. doi: 10.1371/journal.pone.0008578 PMC2798952 20052417

[B54] Hu Y. L. Pang W. Huang Y. Zhang Y. Zhang C. J. (2018). The gastric microbiome is perturbed in advanced gastric adenocarcinoma identified through shotgun metagenomics. Front. Cell. infection Microbiol. 8. doi: 10.3389/fcimb.2018.00433 PMC6299874 30619779

[B55] Huang Y. J. Nariya S. Harris J. M. Lynch S. V. Choy D. F. Arron J. R.. (2015). The airway microbiome in patients with severe asthma: associations with disease features and severity. J. Allergy Clin. Immunol. 136, 874–884. doi: 10.1016/j.jaci.2015.05.044 26220531 PMC4600429

[B56] Huerta A. Soler N. Esperatti M. Guerrero M. Menendez R. Gimeno A.. (2014). ). Importance of Aspergillus spp. isolation in Acute exacerbations of severe COPD: Prevalence, factors and follow-up: The FUNGI-COPD study. Respir. Res. 15. doi: 10.1186/1465-9921-15-17 PMC3996133 24517318

[B57] Huffnagle G. B. Dickson R. P. Lukacs N. W. (2017). The respiratory tract microbiome and lung inflammation: a two-way street. Mucosal Immunol. 10, 299–306. doi: 10.1038/mi.2016.108 27966551 PMC5765541

[B58] Invernizzi R. Wu B. G. Barnett J. Ghai P. Kingston S. Hewitt R. J.. (2021). The respiratory microbiome in chronic hypersensitivity pneumonitis is distinct from that of idiopathic pulmonary fibrosis. Am. J. Respir. Crit. Care Med. 203, 339–347. doi: 10.1164/rccm.202002-0460OC 32692582 PMC7874329

[B59] Jiang X. Zhang Y. Wang H. Wang Z. Hu S. Cao C.. (2022). In-depth metaproteomics analysis of oral microbiome for lung cancer. Research.2022, 9781578. doi: 10.34133/2022/9781578 36320634 PMC9590273

[B60] Jorth P. Turner K. H. Gumus P. Nizam N. Buduneli N. Whiteley M. (2014). Metatranscriptomics of the human oral microbiome during health and disease. MBio 5. doi: 10.1128/mBio.01012-14 PMC3977359 24692635

[B61] Ju F. Zhang T. (2015). 16S rRNA gene high-throughput sequencing data mining of microbial diversity and interactions. Appl. Microbiol. Biotechnol. 99, 4119–4129. doi: 10.1007/s00253-015-6536-y 25808518

[B62] Katsoulis O. Pitts O. R. Singanayagam A. (2024). The airway mycobiome and interactions with immunity in health and chronic lung disease. Oxford Open Immunol. 5, iqae009. doi: 10.1093/oxfimm/iqae009 PMC11357796 39206335

[B63] Kembel S. W. Wu M. Eisen J. A. Green J. L. (2012). Incorporating 16S gene copy number information improves estimates of microbial diversity and abundance. PloS Comput. Biol. 8 doi: 10.1371/journal.pcbi.1002743 PMC3486904 23133348

[B64] Kim Y. G. Udayanga K. G. S. Totsuka N. Weinberg J. B. Núñez G. Shibuya A. (2014). Gut dysbiosis promotes M2 macrophage polarization and allergic airway inflammation via fungi-induced PGE2. Cell Host Microbe 15. doi: 10.1016/j.chom.2013.12.010 PMC3957200 24439901

[B65] Kleiner M. (2019). Metaproteomics: much more than measuring gene expression in microbial communities. MSystems 4. doi: 10.1128/msystems.00115-19 PMC6529545 31117019

[B66] Kogawa M. Hosokawa M. Nishikawa Y. Mori K. Takeyama H. (2018). Obtaining high-quality draft genomes from uncultured microbes by cleaning and co-assembly of single-cell amplified genomes. Sci. Rep. 8. doi: 10.1038/s41598-018-20384-3 PMC5794965 29391438

[B67] Kopylova E. Navas-Molina J. A. Mercier C. Xu Z. Z. Mahé F. He Y.. (2016). Open-source sequence clustering methods improve the state of the art. MSystems 1. doi: 10.1128/msystems.00003-15 PMC5069751 27822515

[B68] Kumpitsch C. Koskinen K. Schöpf V. Moissl-Eichinger C. (2019). The microbiome of the upper respiratory tract in health and disease. BMC Biol. 17. doi: 10.1186/s12915-019-0703-z PMC6836414 31699101

[B69] Kuruvilla M. E. Lee F. E. H. Lee G. B. (2019). Understanding asthma phenotypes, endotypes, and mechanisms of disease. Clin. Rev. Allergy Immunol. 56, 219–233. doi: 10.1007/s12016-018-8712-1 30206782 PMC6411459

[B70] Lai L. A. Tong Z. Chen R. Pan S. (2019). Metaproteomics study of the gut microbiome. Methods Mol. Biol. 1871, 123–132. doi: 10.1007/978-1-4939-8814-3_8 30276736

[B71] Lambring C. B. Siraj S. Patel K. Sankpal U. T. Mathew S. Basha R. (2019). Impact of the microbiome on the immune system. Crit. Rev. Immunol. 39, 313–328. doi: 10.1615/CritRevImmunol.2019033233 32422014 PMC7362776

[B72] Lamichhane S. Sen P. Dickens A. M. Orešič M. Bertram H. C. (2018). Gut metabolome meets microbiome: A methodological perspective to understand the relationship between host and microbe. Methods 149, 3–12. doi: 10.1016/j.ymeth.2018.04.029 29715508

[B73] Leung K. Zahn H. Leaver T. Konwar K. M. Hanson N. W. Pagé A. P.. (2012). A programmable droplet-based microfluidic device applied to multiparameter analysis of single microbes and microbial communities. Proc. Natl. Acad. Sci. United States America 109, 7665–7670. doi: 10.1073/pnas.1106752109 PMC3356603 22547789

[B74] Li R. Li J. Zhou X. (2024). Lung microbiome: new insights into the pathogenesis of respiratory diseases. Sig Transduct Target Ther. 9, 19. doi: 10.1038/s41392-023-01722-y PMC10791971 38228603

[B75] Li S. Yang S. Zhou Y. Disoma C. Dong Z. Du A.. (2021). Microbiome profiling using shotgun metagenomic sequencing identified unique microorganisms in COVID-19 patients with altered gut microbiota. Front. Microbiol. 12. doi: 10.3389/fmicb.2021.712081 PMC8542975 34707577

[B76] Lipinski J. H. Ranjan P. Dickson R. P. O‘wyer D. N. (2024). The lung microbiome. J. Immunol. 212, 1269–1275. doi: 10.4049/jimmunol.2300716 38560811 PMC11073614

[B77] Liu H. Liang Z. Cao N. Yi X. Tan X. Liu Z.. (2021). Airway bacterial and fungal microbiome in chronic obstructive pulmonary disease. Med. Microecology 7. doi: 10.1016/j.medmic.2021.100035

[B78] Long S. Yang Y. Shen C. Wang Y. Deng A. Qin Q.. (2020). Metaproteomics characterizes human gut microbiome function in colorectal cancer. NPJ Biofilms Microbiomes 6. doi: 10.1038/s41522-020-0123-4 PMC7093434 32210237

[B79] Máiz L. Nieto R. Cantón R. de la Pedrosa E. G. G. Martinez-García M.Á. (2018). Fungi in bronchiectasis: A concise review. Int. J. Mol. Sci. 19. doi: 10.3390/ijms19010142 PMC5796091 29300314

[B80] Máiz L. Vendrell M. Olveira C. Girón R. Nieto R. Martínez-García M.Á. (2015). Prevalence and factors associated with isolation of aspergillus and candida from sputum in patients with non-cystic fibrosis bronchiectasis. Respiration 89. doi: 10.1159/000381289 25924628

[B81] Man W. H. De Steenhuijsen Piters W. A. A. Bogaert D. (2017). The microbiota of the respiratory tract: Gatekeeper to respiratory health. Nat. Rev. Microbiol. 15, 259–270. doi: 10.1038/nrmicro.2017.14 28316330 PMC7097736

[B82] Marcy Y. Ouverney C. Bik E. M. Lösekann T. Ivanova N. Martin H. G.. (2007). Dissecting biological “dark matter” with single-cell genetic analysis of rare and uncultivated TM7 microbes from the human mouth. Proc. Natl. Acad. Sci. United States America 104, 11889–11894. doi: 10.1073/pnas.0704662104 PMC1924555 17620602

[B83] Martinsen E. M. H. Eagan T. M. L. Leiten E. O. Haaland I. Husebø G. R. Knudsen K. S.. (2021). The pulmonary mycobiome-A study of subjects with and without chronic obstructive pulmonary disease. . PloS One 16. doi: 10.1371/journal.pone.0248967 PMC8026037 33826639

[B84] McKenney E. S. Kendall M. M. (2016). Microbiota and pathogen “pas de deux”: setting up and breaking down barriers to intestinal infection. Pathog. Dis. 74. doi: 10.1093/femspd/ftw051 PMC5985477 27252177

[B85] McShane P. J. Tino G. (2019). Bronchiectasis. Chest 155, 825–833. doi: 10.1016/j.chest.2018.10.027 30403962

[B86] Molyneaux P. L. Cox M. J. Willis-Owen S. A. G. Mallia P. Russell K. E. Russell A. M.. (2014). The role of bacteria in the pathogenesis and progression of idiopathic pulmonary fibrosis. Am. J. Respir. Crit. Care Med. 190, 906–913. doi: 10.1164/rccm.201403-0541OC 25184687 PMC4299577

[B87] Morris I. R. (1988). Functional anatomy of the upper airway. Emergency Med. Clinics North America 6, 639–669. doi: 10.1016/s0733-8627(20)30518-6 3056703

[B88] Morris A. Beck J. M. Schloss P. D. Campbell T. B. Crothers K. Curtis J. L.. (2013). Comparison of the respiratory microbiome in healthy nonsmokers and smokers. Am. J. Respir. Crit. Care Med. 187, 1067–1075. doi: 10.1164/rccm.201210-1913OC 23491408 PMC3734620

[B89] Mortensen K. L. Johansen H. K. Fuursted K. Knudsen J. D. Gahrn-Hansen B. Jensen R. H.. (2011). A prospective survey of Aspergillus spp. in respiratory tract samples: Prevalence, clinical impact and antifungal susceptibility. Eur. J. Clin. Microbiol. Infect. Dis. 30, 1355–1363. doi: 10.1007/s10096-011-1229-7 21541671

[B90] Mowat E. Rajendran R. Williams C. McCulloch E. Jones B. Lang S.. (2010). Pseudomonas aeruginosa and their small diffusible extracellular molecules inhibit Aspergillus fumigatus biofilm formation. FEMS Microbiol. Lett. 313, 96–102. doi: 10.1111/j.1574-6968.2010.02130.x 20964704

[B91] Mthembu N. Ikwegbue P. Brombacher F. Hadebe S. (2021). Respiratory viral and bacterial factors that influence early childhood asthma. Front. Allergy 2. doi: 10.3389/falgy.2021.692841 PMC8974778 35387053

[B92] Natalini J. G. Singh S. Segal L. N. (2023). The dynamic lung microbiome in health and disease. Nat. Rev. Microbiol. 21, 222–235. doi: 10.1038/s41579-022-00821-x 36385637 PMC9668228

[B93] Nguyen L. D. N. Viscogliosi E. Delhaes L. (2015). The lung mycobiome: An emerging field of the human respiratory microbiome. Front. Microbiol. 6. doi: 10.3389/fmicb.2015.00089 PMC4327734 25762987

[B94] Nilsson R. H. Ryberg M. Kristiansson E. Abarenkov K. Larsson K. H. Köljalg U. (2006). Taxonomic reliability of DNA sequences in public sequences databases: A fungal perspective. PloS One 1. doi: 10.1371/journal.pone.0000059 PMC1762357 17183689

[B95] Nwoko E. S. Q. A. Okeke I. N. (2021). Bacteria autoaggregation: How and why bacteria stick together. Biochem. Soc. Trans. 49, 1147–1157. doi: 10.1042/BST20200718 34110370 PMC8286834

[B96] O’Dwyer D. N. Ashley S. L. Gurczynski S. J. Xia M. Wilke C. Falkowski N. R.. (2019). Lung microbiota contribute to pulmonary inflammation and disease progression in pulmonary fibrosis. Am. J. Respir. Crit. Care Med. 199, 1127–1138. doi: 10.1164/rccm.201809-1650OC 30789747 PMC6515865

[B97] Opron K. Begley L. A. Erb-Downward J. R. Freeman C. Madapoosi S. Alexis N. E.. (2021). Lung microbiota associations with clinical features of COPD in the SPIROMICS cohort. . NPJ Biofilms Microbiomes 7. doi: 10.1038/s41522-021-00185-9 PMC7865064 33547327

[B98] Parameswaran G. I. Wrona C. T. Murphy T. F. Sethi S. (2009). Moraxella catarrhalis acquisition, airway inflammation and protease-antiprotease balance in chronic obstructive pulmonary disease. BMC Infect. Dis. 9. doi: 10.1186/1471-2334-9-178 PMC2780445 19912665

[B99] Peano C. Pietrelli A. Consolandi C. Rossi E. Petiti L. Tagliabue L.. (2013). An efficient rRNA removal method for RNA sequencing in GC-rich bacteria. Microbial Inf. Experimentation 3. doi: 10.1186/2042-5783-3-1 PMC3563489 23294941

[B100] Pei X. M. Yeung M. H. Y. Wong A. N. N. Tsang H. F. Yu A. C. S. Yim A. K. Y.. (2023). Targeted sequencing approach and its clinical applications for the molecular diagnosis of human diseases. Cells 12. doi: 10.3390/cells12030493 PMC9913990 36766834

[B101] Pendleton K. M. Huffnagle G. B. Dickson R. P. (2017). The significance of Candida in the human respiratory tract: Our evolving understanding. Pathog. Dis. 75. doi: 10.1093/femspd/ftx029 PMC6433300 28423168

[B102] Pragman A. A. Kim H. B. Reilly C. S. Wendt C. Isaacson R. E. (2012). The lung microbiome in moderate and severe chronic obstructive pulmonary disease. PloS One 7. doi: 10.1371/journal.pone.0047305 PMC3469539 23071781

[B103] Puiu R. Motoc N. S. Lucaciu S. Ruta M. V. Rajnoveanu R.-M. Todea D. A.. (2024a). The role of lung microbiome in fibrotic interstitial lung disease—A systematic review. Biomolecules 14, 247. doi: 10.3390/biom14030247 38540667 PMC10968628

[B104] Pulvirenti G. Parisi G. F. Giallongo A. Papale M. Manti S. Savasta S.. (2019). Lower airway microbiota. Front. Pediatr. 7. doi: 10.3389/fped.2019.00393 PMC6776601 31612122

[B105] Qin J. Li R. Raes J. Arumugam M. Burgdorf K. S. Manichanh C.. (2010). A human gut microbial gene catalogue established by metagenomic sequencing. Nature 464. doi: 10.1038/nature08821 PMC3779803 20203603

[B106] Quince C. Walker A. W. Simpson J. T. Loman N. J. Segata N. (2017). Shotgun metagenomics, from sampling to analysis. Nat. Biotechnol. 35, 833–844. doi: 10.1038/nbt.3935 28898207

[B107] Quinn R. A. Phelan V. V. Whiteson K. L. Garg N. Bailey B. A. Lim Y. W.. (2016). Microbial, host and xenobiotic diversity in the cystic fibrosis sputum metabolome. ISME J. 10, 1483–1498. doi: 10.1038/ismej.2015.207 26623545 PMC5029181

[B108] Rechenberger J. Samaras P. Jarzab A. Behr J. Frejno M. Djukovic A.. (2019). Challenges in clinical metaproteomics highlighted by the analysis of acute leukemia patients with gut colonization by multidrug-resistant enterobacteriaceae. Proteomes 7. doi: 10.3390/proteomes7010002 PMC6473847 30626002

[B109] Rogers G. B. Zain N. M. M. Bruce K. D. Burr L. D. Chen A. C. Rivett D. W.. (2014). ). A novel microbiota stratification system predicts future exacerbations in bronchiectasis. Ann. Am. Thorac. Soc. 11, 496–503. doi: 10.1513/AnnalsATS.201310-335OC 24592925

[B110] Rozaliyani A. Antariksa B. Nurwidya F. Zaini J. Setianingrum F. Hasan F.. (2023). The fungal and bacterial interface in the respiratory mycobiome with a focus on aspergillus spp. Life 13. doi: 10.3390/life13041017 PMC10142979 37109545

[B111] Sahin-Yilmaz A. Naclerio R. M. (2011). Anatomy and physiology of the upper airway. Proc. Am. Thorac. Soc. 8, 31–39. doi: 10.1513/pats.201007-050RN 21364219

[B112] Saiman L. Siegel J. (2004). Infection control in cystic fibrosis. Clin. Microbiol. Rev. 17, 57–71. doi: 10.1128/CMR.17.1.57-71.2004 14726455 PMC321464

[B113] Salvato F. Hettich R. L. Kleiner M. (2021). Five key aspects of metaproteomics as a tool to understand functional interactions in host-associated microbiomes. PloS Pathog. 17. doi: 10.1371/JOURNAL.PPAT.1009245 PMC7906368 33630960

[B114] Schenck L. P. Surette M. G. Bowdish D. M. (2016). Composition and immunological significance of the upper respiratory tract microbiota. FEBS Lett. 590, 3705–3720. doi: 10.1002/1873-3468.12455 27730630 PMC7164007

[B115] Schmieder R. Edwards R. (2011). Fast identification and removal of sequence contamination from genomic and metagenomic datasets. PloS One 6. doi: 10.1371/journal.pone.0017288 PMC3052304 21408061

[B116] Schneeberger P. H. H. Prescod J. Levy L. Hwang D. Martinu T. Coburn B. (2019). Microbiota analysis optimization for human bronchoalveolar lavage fluid. . Microbiome 7. doi: 10.1186/s40168-019-0755-x PMC6821041 31665066

[B117] Schoch C. L. Seifert K. A. Huhndorf S. Robert V. Spouge J. L. Levesque C. A.. (2012). Nuclear ribosomal internal transcribed spacer (ITS) region as a universal DNA barcode marker for Fungi. Proc. Natl. Acad. Sci. United States America 109, 6241–6246. doi: 10.1073/pnas.1117018109 PMC3341068 22454494

[B118] Segal L. N. Blaser M. J. (2014). A brave new world: The lung microbiota in an era of change. Ann. Am. Thorac. Soc. 11. doi: 10.1513/AnnalsATS.201306-189MG PMC3972973 24437400

[B119] Sethi S. (2000). Infectious etiology of acute exacerbations of chronic bronchitis. Chest 117. doi: 10.1378/chest.117.5_suppl_2.380S 10843981

[B120] Sethi S. Maloney J. Grove L. Wrona C. Berenson C. S. (2006). Airway inflammation and bronchial bacterial colonization in chronic obstructive pulmonary disease. Am. J. Respir. Crit. Care Med. 173. doi: 10.1164/rccm.200509-1525OC PMC2662918 16474030

[B121] Sharpton T. J. (2014). An introduction to the analysis of shotgun metagenomic data. Front. Plant Sci. 5. doi: 10.3389/fpls.2014.00209 PMC4059276 24982662

[B122] Shih C. J. Chen P. Y. Liaw C. C. Lai Y. M. Yang Y. L. (2014). Bringing microbial interactions to light using imaging mass spectrometry. Natural Product Rep. 31, 739–755. doi: 10.1039/c3np70091g 24452118

[B123] Siqueira J. F. Fouad A. F. Rôças I. N. (2012). Pyrosequencing as a tool for better understanding of human microbiomes. J. Oral. Microbiol. 4. doi: 10.3402/jom.v4i0.10743 PMC3266102 22279602

[B124] Soler N. Ewig S. Torres A. Filella X. Gonzalez J. Zaubet A. (1999). Airway inflammation and bronchial microbial patterns in patients with stabl e chronic obstructive pulmonary disease. Eur. Respir. J. 14, 1015–1022. doi: 10.1183/09031936.99.14510159 10596683

[B125] Sultan M. Amstislavskiy V. Risch T. Schuette M. Dökel S. Ralser M.. (2014). Influence of RNA extraction methods and library selection schemes on RNA-seq data. BMC Genomics 15. doi: 10.1186/1471-2164-15-675 PMC4148917 25113896

[B126] Sze M. A. Dimitriu P. A. Hayashi S. Elliott W. M. McDonough J. E. Gosselink J. V.. (2012). ). The lung tissue microbiome in chronic obstructive pulmonary disease. Am. J. Respir. Crit. Care Med. 185, 1073–1080. doi: 10.1164/rccm.201111-2075OC 22427533 PMC3359894

[B127] Takeuchi S. Kawada J.-I. Suzuki A. Sakamoto K. Fukuda Y. Horiba K.. (2025a). Metagenomic analysis of lung microbiome in patients with interstitial lung diseases and sarcoidosis: an experimental study. Health Sci. Rep. 8, e70328. doi: 10.1002/hsr2.70328 39927182 PMC11803077

[B128] Tanca A. Palomba A. Fraumene C. Pagnozzi D. Manghina V. Deligios M.. (2016). ). The impact of sequence database choice on metaproteomic results in gut microbiota studies. Microbiome 4. doi: 10.1186/s40168-016-0196-8 PMC5037606 27671352

[B129] Tangedal S. Nielsen R. Aanerud M. Persson L. J. Wiker H. G. Bakke P. S.. (2019). Sputum microbiota and inflammation at stabl e state and during exacerbations in a cohort of chronic obstructive pulmonary disease (COPD) patients. PloS One 14. doi: 10.1371/journal.pone.0222449 PMC6748569 31527888

[B130] Thorsen J. Stokholm J. Rasmussen M. A. Roggenbuck-Wedemeyer M. Vissing N. H. Mortensen M. S.. (2022). Asthma and wheeze severity and the oropharyngeal microbiota in children and adolescents. Ann. Am. Thorac. Soc. 19, 2031–2043. doi: 10.1513/AnnalsATS.202110-1152OC 35904980

[B131] Tiew P. Y. Dicker A. J. Keir H. R. Poh M. E. Pang S. L. Aogáin M.. (2021). A high-risk airway mycobiome is associated with frequent exacerbation and mortality in COPD. Eur. Respir. J. 57. doi: 10.1183/13993003.02050-2020 32972986

[B132] Tiew P. Y. San Ko F. W. Pang S. L. Matta S. A. Sio Y. Y. Poh M. E.. (2020). Environmental fungal sensitisation associates with poorer clinical outcomes in COPD. Eur. Respir. J. 56. doi: 10.1183/13993003.00418-2020 PMC7453645 32341102

[B133] Tolonen A. C. Xavier R. J. (2017). Dissecting the human microbiome with single-cell genomics. Genome Med. 9, 1–3. doi: 10.1186/s13073-017-0448-7 28615076 PMC5471897

[B134] Tomkovich S. Jobin C. (2016). Microbiota and host immune responses: A love-hate relationship. Immunology 147, 1–10. doi: 10.1111/imm.12538 26439191 PMC4693877

[B135] Tunney M. M. Einarsson G. G. Wei L. Drain M. Klem E. R. Cardwell C.. (2013). Lung microbiota and bacterial abundance in patients with bronchiectasis when clinically stabl e and during exacerbation. Am. J. Respir. Crit. Care Med. 187, 1118–1126. doi: 10.1164/rccm.201210-1937OC 23348972 PMC3734618

[B136] Usyk M. Peters B. A. Karthikeyan S. McDonald D. Sollecito C. C. Vazquez-Baeza Y.. (2023). Comprehensive evaluation of shotgun metagenomics, amplicon sequencing, and harmonization of these platforms for epidemiological studies. Cell Rep. Methods 3. doi: 10.1016/j.crmeth.2022.100391 PMC9939430 36814836

[B137] Valverde-Molina J. García-Marcos L. (2023). Microbiome and asthma: microbial dysbiosis and the origins, phenotypes, persistence, and severity of asthma. Nutrients 15. doi: 10.3390/nu15030486 PMC9921812 36771193

[B138] van Woerden H. C. Gregory C. Brown R. Marchesi J. R. Hoogendoorn B. Matthews I. P. (2013). Differences in fungi present in induced sputum samples from asthma patients and non-atopic controls: A community based case control study. BMC Infect. Dis. 13. doi: 10.1186/1471-2334-13-69 PMC3570489 23384395

[B139] Větrovský T. Baldrian P. (2013). The variability of the 16S rRNA gene in bacterial genomes and its consequences for bacterial community analyses. PloS One 8. doi: 10.1371/journal.pone.0057923 PMC3583900 23460914

[B140] Vidal S. de la Horra C. Martín J. Montes-Cano M. A. Rodríguez E. Respaldiza N.. (2006). ). Pneumocystis jirovecii colonisation in patients with interstitial lung disease. Clin. Microbiol. Infection 12, 231–235. doi: 10.1111/j.1469-0691.2005.01337.x 16451409

[B141] Vissers M. de Groot R. Ferwerda G. (2014). Severe viral respiratory infections: are bugs bugging? Mucosal Immunol. 7, 227–238. doi: 10.1038/mi.2013.93 24220300

[B142] Walker A. W. Martin J. C. Scott P. Parkhill J. Flint H. J. Scott K. P. (2015). 16S rRNA gene-based profiling of the human infant gut microbiota is strongly influenced by sample processing and PCR primer choice. . Microbiome 3. doi: 10.1186/s40168-015-0087-4 PMC4482049 26120470

[B143] Wang Y. Zhou Y. Xiao X. Zheng J. Zhou H. (2020). Metaproteomics: A strategy to study the taxonomy and functionality of the gut microbiota. J. Proteomics 219, 103737. doi: 10.1016/j.jprot.2020.103737 32198072

[B144] Watrous J. D. Phelan V. V. Hsu C. C. Moree W. J. Duggan B. M. Alexandrov T.. (2013). Microbial metabolic exchange in 3D. ISME J. 7, 770–780. doi: 10.1038/ismej.2012.155 23283018 PMC3603389

[B145] Weaver D. Gago S. Bromley M. Bowyer P. (2019). The human lung mycobiome in chronic respiratory disease: limitations of methods and our current understanding. Curr. Fungal Infection Rep. 13, 109–119. doi: 10.1007/s12281-019-00347-5

[B146] Weisskopf L. Schulz S. Garbeva P. (2021). Microbial volatile organic compounds in intra-kingdom and inter-kingdom interactions. Nat. Rev. Microbiol. 19, 391–404. doi: 10.1038/s41579-020-00508-1 33526910

[B147] Wenzel S. E. Schwartz L. B. Langmack E. L. Halliday J. L. Trudeau J. B. Gibbs R. L.. (1999). ). Evidence that severe asthma can be divided pathologically into two inflammatory subtypes with distinct physiologic and clinical characteristics. Am. J. Respir. Crit. Care Med. 160, 1001–1008. doi: 10.1164/ajrccm.160.3.9812110 10471631

[B148] West J. B. (1978). Regional differences in the lung. Chest 74, 426–437. doi: 10.1016/S0012-3692(15)37392-X 699656

[B149] Whiteside S. A. McGinniss J. E. Collman R. G. (2021). The lung microbiome: Progress and promise. J. Clin. Invest. 131. doi: 10.1172/JCI150473 PMC8321564 34338230

[B150] Wilmanski T. Rappaport N. Earls J. C. Magis A. T. Manor O. Lovejoy J.. (2019a). Blood metabolome predicts gut microbiome α-diversity in humans. Nat. Biotechnol. 37, 1217–1228. doi: 10.1038/s41587-019-0233-9 31477923

[B151] Wilmes P. Bond P. L. (2004). The application of two-dimensional polyacrylamide gel electrophoresis and downstream analyses to a mixed community of prokaryotic microorganisms. Environ. Microbiol. 6, 911–920. doi: 10.1111/j.1462-2920.2004.00687.x 15305916

[B152] Xia L. C. Cram J. A. Chen T. Fuhrman J. A. Sun F. (2011). Accurate genome relative abundance estimation based on shotgun metagenomic reads. PloS One 6. doi: 10.1371/journal.pone.0027992 PMC3232206 22162995

[B153] Xie Y. Wu G. Tang J. Luo R. Patterson J. Liu S.. (2014). SOAPdenovo-Trans: *De novo* transcriptome assembly with short RNA-Seq reads. Bioinformatics 30, 1660–1666. doi: 10.1093/bioinformatics/btu077 24532719

[B154] Yang B. Wang Y. Qian P. Y. (2016). Sensitivity and correlation of hypervariable regions in 16S rRNA genes in phylogenetic analysis. BMC Bioinf. 17. doi: 10.1186/s12859-016-0992-y PMC4802574 27000765

[B155] Zhang Y. Wang J. (2023). Cellular and molecular mechanisms in idiopathic pulmonary fibrosis. Adv. Respir. Med. 91, 26–48. doi: 10.3390/arm91010005 36825939 PMC9952569

[B156] Zhao L. Luo J. L. Ali M. K. Spiekerkoetter E. Nicolls M. R. (2023). The human respiratory microbiome current understandings and future directions. Am. J. Respir. Cell Mol. Biol. 68, 245–255. doi: 10.1165/rcmb.2022-0208TR 36476129 PMC9989478

[B157] Zheng D. Liwinski T. Elinav E. (2020). Interaction between microbiota and immunity in health and disease. Cell Res. 30,, 492–506. doi: 10.1038/s41422-020-0332-7 32433595 PMC7264227

[B158] Zierer J. Jackson M. A. Kastenmüller G. Mangino M. Long T. Telenti A.. (2018). ). The fecal metabolome as a functional readout of the gut microbiome. Nat. Genet. 50, 790–795. doi: 10.1038/s41588-018-0135-7 29808030 PMC6104805

